# Molecular insights into the surface-specific arrangement of complement C5 convertase enzymes

**DOI:** 10.1186/s12915-015-0203-8

**Published:** 2015-11-09

**Authors:** Evelien T. M. Berends, Ronald D. Gorham, Maartje Ruyken, Jasper A. Soppe, Hatice Orhan, Piet C. Aerts, Carla J. C. de Haas, Piet Gros, Suzan H. M. Rooijakkers

**Affiliations:** Medical Microbiology, University Medical Center Utrecht, PO G04.614, Heidelberglaan 100, 3584 CX, Utrecht, The Netherlands; Department of Chemistry, Faculty of Science, Crystal and Structural Chemistry, Bijvoet Center for Biomolecular Research, Utrecht University, Utrecht, The Netherlands

**Keywords:** Innate immunity, Inflammatory diseases, Complement, Convertase enzymes, Multi-molecular proteases

## Abstract

**Background:**

Complement is a large protein network in plasma that is crucial for human immune defenses and a major cause of aberrant inflammatory reactions. The C5 convertase is a multi-molecular protease complex that catalyses the cleavage of native C5 into its biologically important products. So far, it has been difficult to study the exact molecular arrangement of C5 convertases, because their non-catalytic subunits (C3b) are covalently linked to biological surfaces through a reactive thioester. Through development of a highly purified model system for C5 convertases, we here aim to provide insights into the surface-specific nature of these important protease complexes.

**Results:**

Alternative pathway (AP) C5 convertases were generated on small streptavidin beads that were coated with purified C3b molecules. Site-specific biotinylation of C3b via the thioester allowed binding of C3b in the natural orientation on the surface. In the presence of factor B and factor D, these C3b beads could effectively convert C5. Conversion rates of surface-bound C3b were more than 100-fold higher than fluid-phase C3b, confirming the requirement of a surface. We determine that high surface densities of C3b, and its attachment via the thioester, are essential for C5 convertase formation. Combining our results with molecular modeling explains how high C3b densities may facilitate intermolecular interactions that only occur on target surfaces. Finally, we define two interfaces on C5 important for its recognition by surface-bound C5 convertases.

**Conclusions:**

We establish a highly purified model that mimics the natural arrangement of C5 convertases on a surface. The developed model and molecular insights are essential to understand the molecular basis of deregulated complement activity in human disease and will facilitate future design of therapeutic interventions against these critical enzymes in inflammation.

**Electronic supplementary material:**

The online version of this article (doi:10.1186/s12915-015-0203-8) contains supplementary material, which is available to authorized users.

## Background

The complement system is a large protein network in plasma that forms the primary host defense barrier against microbial infections [1, 2]. Complement can rapidly label bacterial cells for phagocytosis and generate chemoattractants to recruit immune cells to the site of infection [2]. Furthermore, complement directly kills bacteria via a pore-forming membrane attack complex [3]. Although complement is crucial for local clearance of bacteria at the site of infection, complement activation products (especially C5a) can cause an overwhelming inflammatory response during systemic infections [4]. Furthermore, there is a large list of inflammatory disorders in which erroneous activation of complement on the body’s own cells causes aberrant inflammatory reactions and pathology [5].

**Fig. 1:**
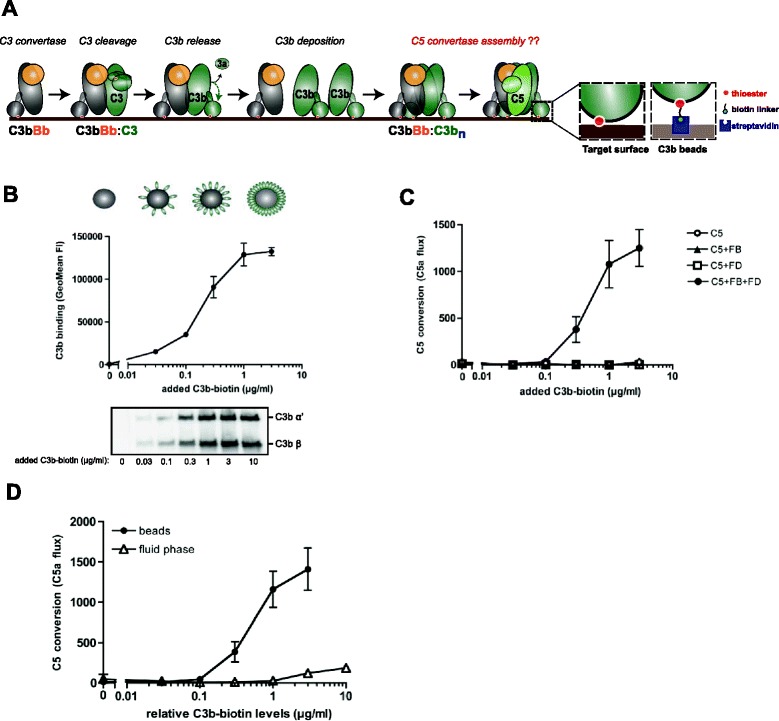
A novel bead-based assay model for purified alternative pathway (AP) C5 convertases. **a** Proposed model for assembly of C5 convertases in the AP. Surface-bound C3 convertase (C3bBb) cleaves multiple C3 molecules into C3b that covalently binds to target surfaces via the reactive thioester (red dot). Association of deposited C3b molecules with the existing C3 convertase gives rise to multimeric complexes (C3b-C3b_n_) that, together with Bb, can convert C5. The precise arrangement of surface-specific C5 convertases is currently unknown. In the novel C5 convertase assay model described in this study, C3b molecules are site-specifically biotinylated via the thioester and loaded on bacteria-sized streptavidin beads (2.8 μm) to mimic their natural density and orientation on target surfaces. **b** Loading of streptavidin beads with biotinylated C3b was analyzed by flow cytometry or immunoblotting (below). **c** C5 convertase activity of C3b-coated beads that were incubated with factor B (FB), factor D (FD) (together needed to form Bb) and C5. Conversion of C5 was determined by measuring release of C5a in the supernatant using a calcium mobilization assay with U937-C5aR cells. Values represent absolute C5a flux (mean fluorescence of stimulated cells subtracted by the mean fluorescence before stimulus). **d** C5 convertase activity of C3b molecules on beads versus C3b molecules in solution. The amount of C3b molecules in solution was adjusted to the levels of C3b loaded onto the beads (relative C3b-biotin levels) and both were incubated with FB, FD and C5. **b**–**d** Data of three independent experiments, presented as means ± standard deviation (SD). Immunoblot is a representative of three independent experiments

Complement proteins circulate in the blood as inactive precursors, but are immediately activated upon contact with target cells. Recognition of target cells occurs via different large molecules (antibodies, lectins) that bind to microbial surface structures and trigger a step-wise activation process in which protein binding and cleavage events occur in a well-defined order [2, 6]. All initiation pathways converge in the formation of short-lived C3 convertase enzymes on the target surface. These C3 convertases cleave the major complement protein C3 into the large, reactive C3b molecule that can covalently attach to target surfaces to label them for rapid opsonization and phagocytosis [7]. In the alternative pathway (AP), the C3 convertase consists of two protein subunits: the non-catalytic protein C3b in complex with protease Bb (C3bBb) (Fig. 1a) [6]. Structural studies suggested that the C3b unit of the C3 convertase forms a dimer with its substrate C3 [8]. Since Bb is bound to a flexible domain in C3b it can swing towards the substrate and cleave the scissile bond in C3. The cleavage results in the release of C3a and, due to large structural rearrangements, the reactive thioester of newly formed C3b becomes exposed and provokes covalent attachment of C3b to any hydroxyl or amine group on the target surface [9, 10]. During the massive labelling of surfaces with C3b, association of one or more C3b molecules to the existing C3 convertase changes the substrate specificity of the enzyme that will then cleave C5 [11, 12]. The exact molecular mechanism for this ‘convertase switch’ is poorly understood. Previous studies have indicated that C3b molecules can adopt a surface-specific conformation [13], but the exact difference between C3b molecules in solution versus a surface remains unknown. For instance the exact stoichiometry and molecular arrangement of C3b molecules on the surface remains unclear. Detailed molecular analyses are largely complicated by the covalent linkage of C3b molecules to the target surface and the requirement of C3 convertases to generate high C3b densities [12]. In this study we present a novel, purified functional assay model for C5 convertases in which we mimic the natural orientation and density of C3b molecules on bead surfaces. Using functional C5 conversion analyses, we provide molecular insights into the surface-specific conformation of C3b molecules. Furthermore, molecular studies highlight two important interfaces for the recognition of substrate C5 by surface-bound convertase enzymes. These increased molecular insights into C5 convertase enzymes are essential to understand the molecular basis for deregulated and excessive convertase activity in human disease and will be critical for future design of therapeutic interventions against the undesired activation of complement during systemic infections and acute inflammatory processes.

## Results

### A novel assay model to analyze purified AP C5 convertases

In order to mimic physiological surface-bound C3b, we labelled purified C3b with biotin via the thioester by activating plasma-purified C3 into C3b in the presence of a biotinylation agent that reacts with the cysteine residue of the C3b thioester (maleimide-PEG2-biotin) [14–16]. These biotinylated C3b molecules were subsequently loaded onto small magnetic streptavidin beads that have a diameter of 2.8 μm (bacteria-sized) and a theoretical binding capacity of around 50,000 C3b molecules per particle (Fig. 1a). We first established that C3b-biotin molecules could bind to streptavidin beads in a dose-dependent manner (Fig. 1b). To assay C5 convertase activity, C3b-loaded beads were incubated with purified factor B (FB), factor D (FD) and C5 after which C5 conversion was quantified by analyzing C5a release into the supernatant using a calcium mobilization assay with C5a receptor (C5aR) transfected cells [17, 18]. We observed effective conversion of C5 in the presence of FB and FD, which are both needed to form protease fragment Bb, which was dependent on the concentration of C3b on the beads. As a control, we showed that C3b beads incubated with C5 alone, C5 + FB, or C5 + FD, did not convert C5 (Fig. 1c). Next, we compared C5 convertase activity of surface-bound convertases (C3b beads) with monomeric C3 convertases in fluid-phase (C3bBb) that are known for their low-level and inefficient conversion of C5 [19]. At equal amounts of C3b, C5 conversion was over 100-fold more efficient on the beads than in solution (Fig. 1d) (C5a in supernatant was quantified by comparing calcium responses with known concentrations of purified C5a; Additional file 1: Figure S1). These data strongly suggest that surface-bound C3b molecules can adopt a unique conformation or arrangement to cleave C5 that does not exist in fluid-phase. Importantly, this reconstituted model is the first system that mimics this critical orientation and density of C3b molecules on a surface using highly purified components and non-covalent surface attachment and is therefore ideal to assess C5 convertase biology.

### C5 binds to C3b-coated beads

**Fig. 2:**
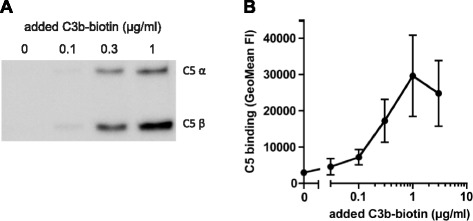
C5 binds to C3b-coated beads. C3b-coated streptavidin beads were incubated with C5 (in the absence of FB and FD). Binding of C5 to C3b beads was determined by **a** Western blotting or **b** flow cytometry. **a** is a representative gel of three independent experiments; **b** shows data of three independent experiments, presented as means ± standard deviation (SD)

According to the literature, an increased density of C3b molecules on serum-opsonized particles creates high-affinity binding sites for C5 [20]. To analyze whether C5 could stably bind to C3b-coated beads, we loaded streptavidin beads with different concentrations of biotinylated C3b and analyzed the binding of C5 by immunoblotting and flow cytometry. To prevent proteolytic cleavage of C5 by Bb, we performed these binding studies in the absence of FB and FD. C5 could specifically bind to C3b on the beads, while no binding was observed on empty beads (Fig. 2a). Flow cytometry studies indicated that the binding of C5 to surface-bound C3b increases at higher concentrations of C3b on beads (Fig. 2b). Using size-exclusion chromatography, we found that this interaction was exclusive for surface-bound C3b since C5 did not detectably bind biotinylated C3b in solution (Additional file 1: Figure S2). These binding studies show that attachment of C3b molecules to a surface alters the binding affinity of C5 for C3b.

### Attachment of C3b with the thioester toward the surface is critical for C5 convertase activity

**Fig. 3:**
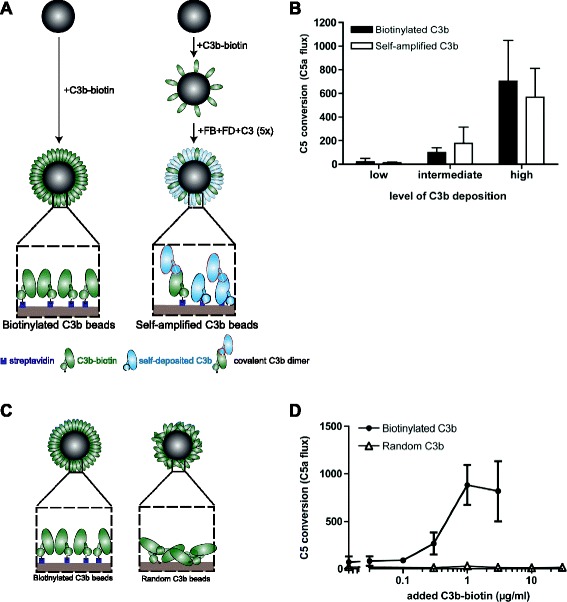
Attachment of C3b with the thioester toward the surface is critical for C5 convertase activity. **a** Left, streptavidin beads with site-specifically biotinylated C3b molecules. Right, self-amplified C3b beads were generated by coating streptavidin beads with a low concentration of C3b-biotin after which FB, FD and C3 were added for five repeating incubations to allow natural deposition of C3b and formation of covalently associated C3b multimers (outlined in red). **b** C5 convertase activity on self-amplified and biotinylated C3b beads. Beads (containing equal levels of C3b) were incubated with FB, FD and C5 and C5a release was determined by calcium mobilization. **c** Random C3b beads were generated by coupling C3b-biotin onto tosyl-activated beads. **d** C5 convertase activity on random and biotinylated C3b beads. (b, d) Data of three independent experiments, presented as means ± standard deviation (SD)

Previous studies have suggested that the deposition of C3b molecules by the C3 convertase is important for generation of efficient C5 convertases. It was proposed that covalent C3b multimers, generated when a newly formed C3b molecule reacts with an existing C3 convertase via the thioester, might be important for C5 convertase activity [21]. In order to investigate this, we generated ‘self-amplified’ C3b beads on which the majority of C3b molecules are deposited via C3 convertases. To this end we first coated beads with low concentrations of C3b-biotin and subsequently performed repeating incubations of the beads with FB, FD and C3 (Fig. 3a). After five rounds of self-amplification, we obtained beads of which 70–90 % of C3b molecules were deposited by a C3 convertase (Additional file 1: Figure S3A). Using Western blotting, we confirmed generation of covalently attached C3b multimers (Additional file 1: Figure S3B) [22]. Next, we compared the C5 conversion by ‘self-amplified C3b’ with ‘biotinylated C3b’ beads that contained equal amounts of C3b, either loaded via the biotin-streptavidin interaction or by C3 conversion. We found no difference in C5 conversion efficiency between beads loaded with self-amplified C3b versus biotinylated C3b (Fig. 3b). Also when self-amplification was performed in human serum, we found no difference in C5 conversion efficiency between serum-derived C3b and biotinylated C3b (Additional file 1: Figure S3C). Next, we analyzed whether the orientation of C3b molecules with the thioester orientated toward the surface is important for surface-dependent C5 conversion. Therefore we compared the C5 conversion rates of ‘biotinylated C3b’ beads with ‘randomly coupled C3b’ beads (Fig. 3c). For random attachment of C3b to a bead, we used tosyl-activated beads (Tosyl) that randomly bind molecules via primary amino or sulfhydryl groups. For a valid comparison with ‘biotinylated C3b’ beads we here used Tosyl streptavidin beads to bind biotinylated C3b. C3b quantification was performed using flow cytometry (Additional file 1: Figure S4). Both beads were analyzed for their capacity to cleave C5. Interestingly, the beads on which C3b was bound in a random orientation completely lacked C5 convertase activity (Fig. 3d), indicating that the orientation of C3b molecules with the thioester group toward the surface is indeed essential for C5 convertase functioning. Altogether these data show that C3b molecules need to bind to the surface in their natural orientation and, in contrast to what was previously postulated, we show that covalent attachment of C3b molecules on top of each other is not required.

### High surface density of C3b is critical for C5 convertase activity

**Fig. 4:**
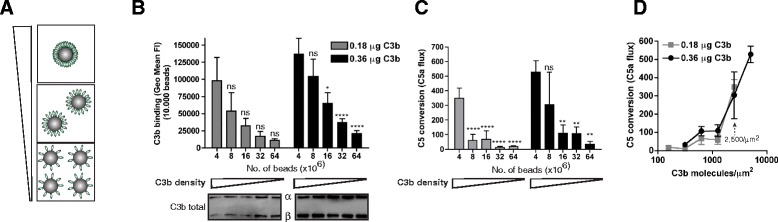
High surface density of C3b is critical for C5 convertase activity. **a** Preparation of beads with different densities of C3b. **b** Mixing a fixed amount of C3b molecules with increasing numbers of streptavidin beads results in lower C3b densities per bead (top, flow cytometry) while total levels of C3b per sample are equal (below, immunoblot). **c** C5 convertase activity on streptavidin beads with different C3b densities. **d** C5 convertase activity plotted against the absolute number of C3b molecules per μm^2^ (calculated from the results in **c**). **b**–**d** Data of three independent experiments, presented as means ± standard deviation (SD). Immunoblot graphs are representative of three independent experiments. Measures of statistical significance were determined by one-way ANOVA for the different amounts of beads versus 4 × 10^6^ beads and displayed as: ns; *P <0.05; **P <0.01; ***P <0.005; and ****P <0.001

Next, we wondered whether the unique surface-specific conformation of C3b molecules could be explained by the fact that a surface allows C3b molecules to come together at a high density. Such a high density may allow for certain intermolecular interactions between C3b molecules that do not occur at a lower density. So far, it has not been possible to functionally compare C3b molecules at different surface densities. Using the streptavidin bead model described here, we could now generate beads with different C3b densities in a well-controlled manner. Similar to the C3b dose–response experiment in Fig. 1b, we incubated 4 × 10^6^ beads with 0.18 μg or 0.36 μg of C3b-biotin. This results in loading of beads with C3b at concentrations below saturation (0.18 μg or 0.36 μg correspond to 0.3 μg/ml and 0.6 μg/ml C3b-biotin in Fig. 1b). Then, we lowered C3b densities by increasing the number of beads (up to 64 × 10^6^ per sample) while keeping the amounts of C3b per sample constant (Fig. 4a, b). Flow cytometric quantification indicated that we successfully reduced the C3b levels per bead in a step-wise manner (Fig. 4b). Using immunoblotting we showed that the total C3b levels in the sample (all beads) was equal (Fig. 4b, Western blots). Then we compared the C5 convertase activity on these beads and found that C5 conversion rates significantly decrease with lower C3b densities (Fig. 4c). Plotting the C5a levels against the absolute numbers of C3b molecules per μm^2^, suggests the requirement of a critical surface density of C3b to trigger effective C5 conversion. In our model, this critical density is reached at concentrations of 2,500 C3b molecules per μm^2^ (Fig. 4d). Altogether these studies suggest that the density of C3b molecules on a surface is a critical determinant for generating effective C5 convertases.

### Inhibitors reveal two important interaction sites for C5 with surface-bound C3b

**Fig. 5:**
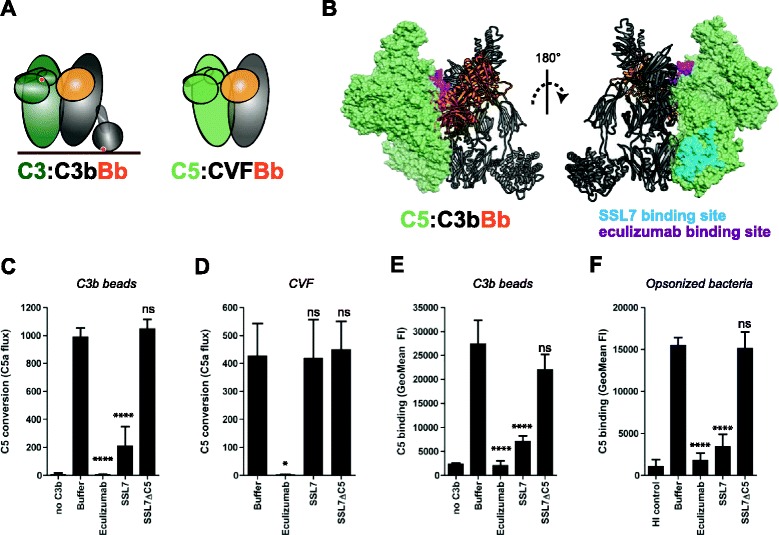
Inhibitors reveal two important interaction sites for C5 with surface-bound C3b. **a** Left, schematic representation of the proposed interaction between substrate C3 and the alternative pathway (AP) C3 convertase (based on crystal structure [8]). Right, binding of C5 to CVFBb (based on the CVF:C5 crystal structure [24]). CVF is a potent C3b homologue that lacks the thioester domain and forms stable C5 convertases in solution. **b** Structural model of the previously proposed AP C5 convertase. The C3/C5 convertase (C3bBb) is shown in ribbon representation, with C3b in gray and Bb in orange, respectively. C5 (green) is shown as a molecular surface, with residue involved in eculizumab (magenta) and SSL7 (cyan) binding colored on the surface. The left and right representations represent the same complex rotated 180° about the vertical axis. **c** C5 conversion on C3b-coated beads in the absence or presence of 20 μg/ml C5 inhibitors (SSL7, SSL7ΔC5, eculizumab) as determined by calcium mobilization of U937-C5aR cells. **d** C5 conversion by soluble CVFBb in the absence or presence of 20 μg/ml C5 inhibitors. **e** C5 binding to C3b-coated beads (loaded with 1 μg/ml C3b-biotin) in absence or presence of 20 μg/ml C5 inhibitors, determined by flow cytometry. **f** C5 binding to pre-opsonized bacteria in absence or presence of 20 μg/ml C5 inhibitors (flow cytometry). **c**–**f** Data of three independent experiments, presented as means ± standard deviation (SD). Measures of statistical significance were determined by one-way ANOVA for the various inhibitors versus buffer control alone and displayed as: ns; *P <0.05; **P <0.01; ***P <0.005; and ****P <0.001

Although the molecular organization of AP C5 convertases is largely unknown, the three-dimensional structure of the alternative pathway C3 convertase (C3bBb) in complex with the staphylococcal complement inhibitor (SCIN) has been determined [8]. This structure suggested that the C3b unit of the C3 convertase forms a head-to-head dimer with its substrate C3 and subsequently allows Bb, bound to a flexible domain in C3b, to swing towards the C3 substrate and cleave the scissile bond (Fig. 5a) [8]. Due to the high structural similarity between C5 and C3 [23], a similar substrate-convertase model was proposed for C5 convertases [24]. Also, the crystal structure of the C3b homologue cobra venom factor (CVF) bound to C5 indicated that the interface between CVF and C5 is highly similar to the C3b-C3 interface in the C3 convertase structure (via domains MG4 and MG5; Fig. 5a) [24]. To study this hypothesis, we first generated a hypothetical model of C3bBb-C5, by overlaying the structures of C3bBb [8] with CVF-C5 (Fig. 5b) [24]. Then, we performed inhibitor analyses in our functional C5 convertase model to investigate the physiological relevance of this proposed C5-C3b interaction. To this end we used eculizumab (Soliris), a humanized antibody against C5 [25], that binds to an epitope within the MG7 domain [26] and would cause steric hindrance of C5 binding to C3b in the proposed model (Fig. 5b). Indeed, we observe that eculizumab potently interferes with C5 conversion, both by surface-bound C3b on beads and soluble CVFBb (Fig. 5c, d). Then, we studied the bacterial protein SSL7 that potently binds C5 and prevents C5 conversion on biological surfaces (bacteria and erythrocytes) [18, 24, 27]. The SSL7-C5-CVF structure revealed that SSL7 binds C5 in a region that would not sterically hinder formation of the proposed C3b-C5 interface (Fig. 5b). Interestingly, we observed that SSL7 inhibited C5 conversion by C3b-coated beads while a mutant of SSL7 defective of C5 binding (SSL7ΔC5, D147K mutant [28]) could not (Fig. 5c). In concordance with the finding that SSL7 can still bind to CVF-C5, we found that SSL7 could not block C5 conversion by CVFBb (Fig. 5d). Combining the results for eculizumab and SSL7 indicates that the interaction sites of both inhibitors are important for the interaction of C5 with surface-bound C3b. To further confirm, we analyzed whether eculizumab and SSL7 could block the binding of C5 to surface-bound C3b. Indeed, we found that both inhibitors could prevent binding of C5 to C3b beads (Fig. 5e). Also, when we analyzed binding of C5 to serum-opsonized bacteria (coated with naturally deposited C3b molecules) we observed that both inhibitors disturb binding of C5 to C3b (Fig. 5f). Altogether these findings indicate that the interaction of C5 with surface-bound C3b occurs at multiple interfaces, including the proposed C5 interaction site similar to the reported CVF-C5 interface (Fig. 5a) and the SSL7-binding site in C5 (Fig. 5b).

## Discussion

A long-standing question in complement biology is how a convertase enzyme can switch between its two substrates, C3 and C5. While the molecular mechanisms underlying C3 conversion are well understood, research on the C5 convertases has been hampered by their ‘surface-specific’ configuration. Although most convertase research has been performed in highly complex protein environments like human plasma, studies performed in semi-purified conditions have significantly improved our understanding of C5 convertases [11, 12, 20, 29, 30]. There it was shown that C5 convertases are specifically formed on target surfaces after a C3 convertase enzyme deposits one or more C3b molecules. Both the conversion efficiency and C5 binding affinity increased when high densities of C3b were present on a biological surface [20, 31]. However, since C3 convertase-dependent C3b deposition was the only method to generate such high densities on a surface, it remained unclear how C3b molecules were arranged and oriented within the C5 convertase. Using the highly purified assay system developed in this study, we here provide detailed insights into the surface-specific molecular arrangement of C5 convertases.

**Fig. 6:**
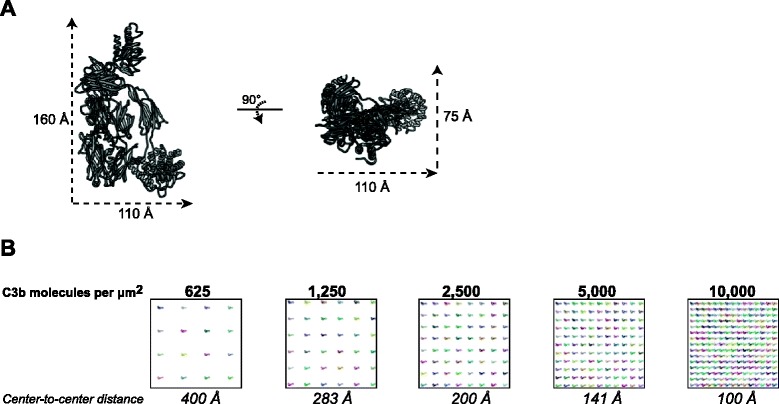
Molecular model for C3b density. **a** The crystallographic structure of C3b [PDB:2I07]. **b** Model for C3b density on a 150 × 150 nm area representing a portion of the bead surface. For each C3b density, the number of molecules per 150 × 150 nm surface area was calculated, and intermolecular distance was calculated assuming a uniform distribution of molecules. C3b molecules were then placed such that the center-to-center distance was equal to the calculated intermolecular distance. C3b is oriented such that the long axis is perpendicular to the surface and the thioester (and in turn the biotin linker) was in contact with the surface

Different theories have been proposed for why C3b molecules on a surface are more effective in forming a C5 convertase. For instance, it was suggested that unique covalent C3b multimers are formed when a newly formed C3b molecule reacts with the C3b molecule of the pre-existing convertase via its released thioester [21, 32]. In our system, we found no evidence for the role of such covalent C3b multimers in C5 conversion. Our findings strongly indicate that surfaces enable the site-specific attachment and high density of C3b molecules that are critical for formation of C5 convertases in the alternative pathway. On our streptavidin beads, we find that C3b concentrations of 5,000 molecules/μm^2^ and higher are needed for effective C5 convertase activity (Fig. 4c). Likely, C3b molecules need to come close to each other to allow C5 binding. To get an idea of the intermolecular distance of C3b molecules on a surface we attempted to model C3b surface density at a molecular level. Based on the known dimensions of a C3b molecule [PDB: 2I07] (Fig. 6a) [7], we placed different numbers of C3b molecules in a hypothetical 150 × 150 nm area representing a portion of the bead surface. The crystallographic structure of C3b was oriented such that the long axis was perpendicular to the surface and the thioester (and in turn the biotin linker) was in contact with the surface (Fig. 6b). For the analyzed C3b densities (Fig. 4c), the number of molecules per 150 × 150 nm surface area was calculated, and intermolecular distance was calculated assuming a uniform distribution of molecules. The C3b molecules were then placed such that the center-to-center distance was equal to the calculated intermolecular distance (Fig. 6b). Based on this model, it seems obvious that a concentration of 1,250 molecules/μm^2^ is too low to allow C3b molecules to make direct contact, or to ‘sandwich’ a C5 molecule (105 Å × 130 Å × 80 Å). Such a ‘sandwich’ model, in which a C5 molecule is captured in between two C3b molecules, seems more likely at a density of 2,500 molecules/μm^2^. However, since C5 conversion rates clearly increase at higher densities (Fig. 4c), the C3b molecules might actually need to make direct contact (like in the model for 10,000 C3b molecules/μm^2^) (Fig. 6c). In comparison, a density of 10,000 C3b molecules/μm^2^ is equivalent to a concentration of 1 mM C3b molecules in fluid-phase (calculated using a 1 μm × 1 μm area on a surface and using the height of a C3b molecule (0.016 μm), assuming that C3b molecules stand up straight due to thioester surface attachment). Such a concentration is unlikely to be reached in vivo since normal plasma levels of C3 are 5 μM. Thus, complement activation on surfaces facilitates high local concentrations of C3b unattainable in fluid-phase. Likely, this explains why others and we have failed to demonstrate binding between C3b and C5 in solution. Next to high surface density, it is still possible that a single C3b molecule adopts a different conformation in solution than when it is fixed to a surface. The TED of C3b is connected to the body of the C3b molecule via its CUB domain [7]. Recent studies indicated that the TED region is very flexible and gives the C3b molecule conformational variability that might potentially be altered when TED is fixed to a surface [33–35]. While we observe that the orientation of C3b molecules (via linkage of TED to a surface) is critical for C5 convertase formation, our studies cannot exclude that the conformation of single C3b molecules attached to a surface is different from their conformation in solution. However, as our studies indicate that orientation of the C3b molecules on the surface is important for C5 convertase formation, the random distribution of C3b molecules in solution likely disfavors critical C3b-C3b or C3b-C5 interactions. A critical note should be placed on these models, since the distribution of C3b molecules on our beads is probably less uniform than depicted. Since we make use of beads that are loaded with streptavidin molecules that, in principle, have four biotin binding sites, the C3b molecules on our beads might actually be closer together. Also, it still remains to be determined how these numbers compare to C3b molecules on a natural biological surface where the thioester is anchored to surface-bound structures (polysaccharides, proteins) [9] that would allow more flexibility and enhance intermolecular contacts. Furthermore, under physiological conditions, the C3 convertase enzymes drive the deposition of C3b molecules close to the surface and it has been proposed that nascent C3b molecules deposit on the surface within a 600 Å radius around the C3 convertase, creating hotspots with C3b molecules at a high density [11]. Probably, the densities that we create on streptavidin beads are very close to the C3b densities created by C3 convertases.

At least 30 different diseases can be traced to unwanted complement activation and there is an urgent medical need for improved treatments [2]. For many of these, the C5 convertase is considered an ideal target for complement therapy; blocking this protease would prevent formation of the major inflammatory trigger C5a but leave phagocytosis of bacteria intact [5]. Also, since these protease complexes are exclusively formed on target cells, targeting the C5 convertase would allow a much more specific and localized treatment than inhibition of C5 in solution. Since eculizumab, the first complement drug now used in the clinic, does not specifically block C5 activation on target cells, its inhibitory effects on solution phase C5 may predispose patients to serious meningococcal infections [36]. Detailed molecular understanding and highly purified assay systems, like we describe in this study, will aid in the development of more specific therapeutic inhibitors targeting the C5 convertase. Furthermore, our newly developed system also opens up new avenues of molecular research to understand how convertase can be regulated. There are a number of suggested C5 convertase regulatory proteins, evolved in humans [37–39] and bacteria [14], for which the mode of action is unclear due to the lack of specific convertase models. We here characterize the mode of action of two potent C5 inhibitors, eculizumab and SSL7. Despite being effective C5 conversion blockers in plasma, it was never clear how these molecules function since they bind C5 at an interface that is distinct from the scissile bond [24, 25]. Now we show for the first time that both inhibitors prevent binding of C5 to surface-bound convertases. Since the epitopes of eculizumab and SSL7 are on complete distant parts of the C5 molecule (Fig. 5b), these data strongly suggest that C5 has two distant sites involved in the interaction with multiple C3b molecules immobilized on surfaces. Interestingly, the inhibitory analyses also show that C5 convertases on C3b beads are distinct from fluid-phase CVF convertases, but similar to naturally occurring C3b-dependent C5 convertases on bacterial surface.

## Conclusions

(i) We establish a novel model system that allows functional and biochemical characterization of surface-specific C5 convertase enzymes.
(ii) We demonstrate that high surface densities of C3b molecules are essential for the formation of C5 convertase complexes.
(iii) We highlight important interfaces for the recognition of substrate C5 to surface-bound convertases.
(iv) The developed model system opens up new areas of biomolecular research to understand hitherto unexplained mechanisms of convertase regulation.
(v) The provided insights and our newly developed model system allows new approaches of pharmaceutical research aimed at developing inhibitors of C5 convertases, which are important therapeutic targets in many inflammatory diseases.

## Methods

### Complement proteins

C3 was isolated from freshly prepared human plasma as described [40]. For C5 isolation, a 1 ml N-hydroxysuccinimide (GE Healthcare, Little Chalfont, UK) column was loaded with the soft tick complement inhibitor OmCI that efficiently binds C5 [23]. OmCI was kindly provided by Miles Nunn, Susan Lea and Matthijs Jore. The OmCI used in this study refers to pOmCI, a double mutant (N78Q/N102Q), which was expressed in *Pichia methanolica* and purified to homogeneity as described [41]. The serum was diluted 1:1 with PBS, 10 mM EDTA was added, and loaded onto the OmCI-coupled column to allow C5 binding to the column. C5 was eluted with 0.1 M ethanolamine, 0.05 M NaCl (pH 11) that was immediately neutralized with 0.1 M HCl in 1 M phosphate, 10 mM NaCl buffer (pH 7.4). Fractions were analyzed by SDS-PAGE and dialyzed against PBS overnight at 4 °C. Activity was confirmed by CH50 analyses in depleted serum (Complement Technology, Inc., Tyler, TX, USA) and compared with commercially obtained C5 from Complement Technology, Inc. FB and FD were expressed in HEK293 cells stably expressing EBNA1 (HEK293E) as described [42] (U-Protein Express, Utrecht, The Netherlands). FB contained an N-terminal His-tag and was isolated from the expression medium via immobilized metal affinity chromatography (HiTrap chelating column, GE Healthcare). FD was isolated by size-exclusion chromatography (Superdex75, GE Healthcare).

### C5 inhibitors

Eculizumab was ordered via the pharmacy and kindly provided by Genmab (Utrecht, The Netherlands). SSL7 and the mutant of SSL7 that cannot bind C5 (SSL7ΔC5) were cloned and expressed in *Escherichia coli* and purified as described previously [18]. The SSL7ΔC5 has the D147K mutation as described [28].

### Biotinylation of C3b

Plasma-purified C3 was activated into C3b in the presence of a biotinylation agent that reacts with the cysteine residue of the C3b thioester as described previously [14–16] with some adaptations. A concentration of 1 mg/ml C3 was activated with 1.1 μg/ml trypsin, for 10 min (min) at 37 °C, in the presence of 100 μg/ml maleimide-PEG2-biotin (Thermo Scientific Pierce Protein Research, Rockford, IL, USA). The reaction was stopped by adding 5.5 μg/ml soybean trypsin inhibitor (SBTI) after which 20 mM iodoacetamide was added and incubated for 30 min on ice. The sample was 1:1 diluted into 20 mM phosphate, 10 mM NaCl buffer (pH 7.4) and applied to a 1 ml MonoQ anion-exchange column (GE Healthcare). C3b-biotin was eluted by a gradient to 55 % 20 mM phosphate, 500 mM NaCl buffer (pH 7.4) using AKTA FPLC (GE Healthcare). Fractions were analyzed by SDS-PAGE and successful biotinylation of the alpha chain was confirmed by Western blotting.

### Preparation of C3b-coated beads

Streptavidin-coated magnetic beads (Dynabeads M-270 Streptavidin, Invitrogen (Carlsbad, CA, USA), 2.8 μm diameter) were washed twice and suspended in VBS-T^+^ (veronal buffered saline: 2 mM veronal, 145 mM NaCl, pH 7.4 (VBS, pH 7.4) containing 2.5 mM MgCl_2_ and 0.05 % Tween). Beads (4 × 10^6^) were loaded with different concentrations of C3b-biotin by incubating a volume of 200 μl beads with 200 μl C3b-biotin (final concentrations as indicated) for 1 h at 4 °C under shaking conditions. For quantification of C3b on beads, pellets were incubated with FITC-conjugated C3 antibody (Protos Immunoresearch, Burlingame, CA, USA) for 30 min at 4 °C under shaking conditions. Beads were washed and analyzed by flow cytometry using a FACSVerse flow cytometer (Becton Dickinson, San Jose, CA, USA). Beads (total of 10,000 events) were gated based on forward/side scatter and the geometric mean fluorescence of the gated population was analyzed using FlowJo software.

### C5 convertase activity assay

C3b-coated beads were incubated with 20 μg/ml C5, 50 μg/ml FB and 5 μg/ml FD in absence or presence of inhibitory proteins (20 μg/ml eculizumab, SSL7, SSL7ΔC5) in a total volume of 100 μl VBS-T^+^ for 1 h at 37 °C while shaking constantly. After incubation, supernatants were collected and analyzed for the presence of C5a. Fluid-phase C5 convertase activity was assayed by incubating C3b-biotin together with 20 μg/ml C5, 50 μg/ml FB and 5 μg/ml FD in VBS-T^+^. CVF-based C5 convertase assays were performed in a total volume of 100 μl in VBS^+^-0.1 % BSA in which 0.01 μg/ml CVF was incubated with 20 μg/ml C5, 50 μg/ml FB and 5 μg/ml FD, in the absence or presence of inhibitory proteins (20 μg/ml eculizumab, SSL7, SSL7ΔC5) for 1 h at 37 °C. The release of C5a in supernatants was determined in a calcium mobilization assay [43]. Stably transfected U937-C5aR cells were loaded with 2 μM Fluo-3-AM (Invitrogen), washed and re-suspended in RPMI/0.05 % HSA to a concentration of 1 × 10^6^ cells/ml. The transient increase in free intracellular calcium concentration was measured by flow cytometry. Cells were gated based on scatter properties. The basal fluorescence level was monitored for 8 s, then stimulus (purified C5a or supernatants of convertase assays) was added and the sample tube was rapidly placed back to the sample holder and the fluorescence measurement continued up to 1 min. Absolute calcium mobilization (C5a flux) was calculated by subtracting background fluorescence from the fluorescence after stimulation. Standard curves were generated using different concentrations of purified C5a (10^−11^–10^−7^ M, Bachem, Bubendorf, Switzerland).

### C5 binding to C3b beads

For analysis of C5 binding, C3b-coated beads were incubated with 50 μl 20 μg/ml C5 in absence or presence of inhibitory proteins (20 μg/ml eculizumab, SSL7, SSL7ΔC5) for 1 h at 37 °C. After washings in PBS-1 % BSA, bead pellets were incubated with 3 μg/ml rabbit anti-C5 (Dako, Carpinteria, CA, USA) in 50 μl PBS-1 % BSA for 45 min at 4 °C. After washing twice, pellets were incubated with FITC-conjugated goat anti-rabbit IgG (1:50, Sigma-Aldrich, St Louis, MO, USA) in 50 μl PBS-1 % BSA for 45 min at 4 °C.

### Western blotting

Bead samples were heated at 95 °C for 5 min in 2× sample buffer (2 % SDS, 20 % glycerol, 20 mM Tris–HCl, pH 6.8 and 1 mg/ml bromophenol blue) with 50 mg/ml dithiothreitol (DTT). A volume of 10 μl of each sample was analyzed by SDS-PAGE (10 %) and electrophoretically transferred to a polyvinylidene difluoride (PVDF) membrane (EMD Millipore, Billerica, MA, USA). Membranes were blocked with 4 % dried skim milk (ELK, Campina. Amersfoort, The Netherlands) in PBS-0.05 % Tween and incubated with peroxidase (PO)-conjugated streptavidin (to analyze C3b-biotinylation) or probed with goat antiserum against human C5 (1:300, Complement Technology, Inc.). The membranes were washed, incubated with PO-conjugated donkey anti-goat IgG (Bio-Connect, Huissen, The Netherlands) and developed by enhanced chemiluminescence (ECL, Fisher Emergo, Landsmeer, The Netherlands).

### C5 binding to pre-opsonized bacteria

To generate pre-opsonized bacteria, *E. coli* MG1655 were grown to midlog phase (OD_660_ ~ 0.5) in lysogeny broth (LB) and suspended in VBS containing 0.1 % BSA and 2.5 mM MgCl_2_ (VBS^+^). Bacteria (~5 × 10^8^ cfu/ml) were pre-incubated for 1 h at 37 °C in 10 % C5 depleted serum (Complement Technology, Inc.), or 10 % normal serum that was heated at 56 °C for 30 min to eliminate complement activity as a control (HI serum). To allow decay of Bb, bacteria were incubated in PBS for 1.5 h at 37 °C. Then, bacteria were washed in VBS^+^ and incubated with 20 μg/ml C5 for 1 h at 37 °C under shaking conditions. After subsequent washing, binding of C5 was detected by flow cytometry. Bacterial cells (total of 10,000 events) were gated based on forward/side scatter and the geometric mean fluorescence of the gated population was analyzed using FlowJo software.

### Preparation of differently coated C3b beads

To generate ‘self-amplified’ C3b beads, streptavidin-coated beads (Dynabeads M-270) were loaded with 0, 0.01 or 0.1 μg/ml C3b-biotin after which the bead pellets were incubated with 20 μg/ml FB and 5 μg/ml FD in a total volume of 125 μl for 10 min at 37 °C under shaking conditions. To prevent fluid-phase C3 conversion, 10 mM EDTA was added, and then 50 μg/ml C3. The beads were washed and the procedure was repeated for 5 rounds in total to allow self-amplification to completion. The C3b levels deposited on the beads were quantified by flow cytometry as described above (FITC-conjugated C3 antibody) and categorized in ‘low’ (for beads with 0 μg/ml C3b-biotin before amplification), ‘intermediate’ (0.01 μg/ml C3b-biotin before amplification) and ‘high’ (0.1 μg/ml C3b-biotin before amplification). Based on flow cytometry quantification, the ‘low’, ‘intermediate’ and ‘high’ self-amplified beads were then compared with beads loaded with 0 μg/ml, 0.3 μg/ml and 3 μg/ml C3b-biotin in a C5 convertase activity assay. For natural amplification in serum, C3b-coated beads were coated with 0.01 μg/ml C3b-biotin after which the beads were incubated in different concentrations of normal pooled human serum (as described [44]) in VBS containing 2.5 mM MgCl_2_ (VBS^+^).

To generate beads bearing C3b molecules in a random orientation, we used two types of tosyl-activated beads: Dynabeads® M-280 Tosylactivated and Dynabeads® M-280 Streptavidin, Invitrogen, 2.8 μm diameter. Both bead types were coated with C3b-biotin in PBS-0.05 % Tween overnight at 37 °C. After washing, beads were analyzed in the C5 convertase activity assay as described above.

To compare beads with different C3b densities but leaving total C3b level in the sample constant, we loaded different amounts of streptavidin-coated beads with 0.18 or 0.36 μg C3b-biotin in a total volume of 600 μl buffer. After washing, 1/3 of loaded beads were incubated with anti-C3 antibodies for flow cytometry analyses or Western blotting while 2/3 of beads were analyzed for C5 convertase activity as described above.

### Molecular modeling

All molecular models were generated using UCSF Chimera [45]. Convertase models were generated based on structures of the C3bBb-SCIN complex [PDB: 2WIN] [8] and the C5-CVF-SSL7 complex [PDB: 3PRX] [24]. The coordinates of the C3b molecule from the C3bBb structure were superimposed on those of CVF from the C5-CVF-SSL7 structure, to model the hypothetical monomeric C3bBb convertase in complex with C5. The SSL7 binding site residues were selected as C5 residues within 6 Å of SSL7 from the crystallographic structure, and the eculizumab binding site residues were chosen based on the known C5 epitope [26]. To examine C3b surface density at a molecular level, a 150 × 150 nm area representing a portion of the bead surface was modeled. The crystallographic structure of C3b [PDB: 2I07] [7] was oriented such that the long axis was perpendicular to the surface and the thioester (and in turn the biotin linker) was in contact with the surface. For each C3b density, the number of molecules per 150 × 150 nm surface area was calculated, and intermolecular distance was calculated assuming a uniform distribution of molecules. C3b molecules were then placed such that the center-to-center distance was equal to the calculated intermolecular distance.

### Statistical analyses

Statistical analyses were performed using Prism (GraphPad Software).

### Supporting data

The data sets supporting the results of this article are available in Additional file 1.

## Additional file

Additional file 1:
**Molecular insights into the surface-specific arrangement of complement C5 convertase enzymes.** **Figure S1.** C5a standard curve for calcium mobilization assay. Different concentrations of purified C5a were added to U937-C5aR cells as stimulus to detect calcium mobilization. Data represent mean values of fluorescence after stimulus, subtracted by the mean fluorescence before stimulus of two independent experiments (presented as means ± standard deviation (SD)). **Figure S2.** Size exclusion chromatography of soluble C3b and C5. C3b-biotin (300μg/ml), C5 (150μg/ml) or a mixture of both proteins at the same concentrations were incubated for 1 hour at 4°C in VBS with 2.5mM MgCl2 and subsequently analysed on a Superdex 200 10/300 GL column (100μl injection volume). Representative chromatogram of three independent experiments. **Figure S3.** **A.** C3b quantification of self-amplified beads. C3b deposition was quantified using a FITC-conjugated C3 antibody and flow cytometry. Black bars indicate the initial biotinylated C3b on beads. White bars indicated the C3 fluorescence that was loaded via self-amplification after 5 rounds of incubation with FB, FD and C3. Total surface C3b levels were categorized in ‘low’, ‘intermediate’, and ‘high’ levels. 'Self' deposited C3b molecules represented 70-90% of the total surface bound C3b molecules. **B.** Western blot analyses of biotinylated and selfamplified C3b on streptavidin beads. As described in Barrio et al, we found evidence for covalent attachment of C3b molecules on top of other C3b (covalent C3b multimers). **C.** C5 convertase activity on C3b beads and serum-amplified C3b beads (with equal amounts of C3b per bead). Data of three independent experiments, presented as means ± SD. **Figure S4.** Quantification of C3b-biotin bound to tosyl streptavidin beads ('biotinylated C3b) versus tosyl-activated beads ('random C3b'). C3b levels were detected by flow cytometry after incubation of beads with an antibody against C3b. (PDF 1672 kb)

## Abbreviations

ANOVA: analysis of variance
AP: alternative pathway
BSA: bovine serum albumin
C5aR: C5a receptor
CVF: cobra venom factor
DTT: dithiothreitol
ECL: enhanced chemiluminescence
FB: Factor B
FD: Factor D
FITC: fluorescein isothiocyanate
IgG: Immunoglobulin G
LB: lysogeny broth
OD: optical density
PBS: phosphate-buffered saline
PO: peroxidase
PVDF: polyvinylidene difluoride
SBTI: soybean trypsin inhibitor
SCIN: staphylococcal complement inhibitor
SD: standard deviation
SDS-PAGE: sodium dodecyl sulfate polyacrylamide gel electrophoresis
SSL7: staphylococcal superantigen-like 7 protein
TED: thioester domain
VBS: veronal buffered saline
