# Supplementary material for: Molecular insights into the surface-specific arrangement of complement C5 convertase enzymes
Source: BMC Biol. 2015 Nov 9;13:93. doi: 10.1186/s12915-015-0203-8 (PMC4638095; doi:10.1186/s12915-015-0203-8)
Supplement: Additional file 1: — Molecular insights into the surface-specific arrangement of complement C5 convertase enzymes. Figure S1. C5a standard curve for calcium mobilization assay. Different concentrations of purified C5a were added to U937-C5aR cells as stimulus to detect calcium mobilization. Data represent mean values of fluorescence after stimulus, subtracted by the mean fluorescence before stimulus of two independent experiments (presented as means ± standard deviation (SD)). Figure S2. Size exclusion chromatography of soluble C3b and C5. C3b-biotin (300μg/ml), C5 (150μg/ml) or a mixture of both proteins at the same concentrations were incubated for 1 hour at 4°C in VBS with 2.5mM MgCl2 and subsequently analysed on a Superdex 200 10/300 GL column (100μl injection volume). Representative chromatogram of three independent experiments. Figure S3. A. C3b quantification of self-amplified beads. C3b deposition was quantified using a FITC-conjugated C3 antibody and flow cytometry. Black bars indicate the initial biotinylated C3b on beads. White bars indicated the C3 fluorescence that was loaded via self-amplification after 5 rounds of incubation with FB, FD and C3. Total surface C3b levels were categorized in ‘low’, ‘intermediate’, and ‘high’ levels. 'Self' deposited C3b molecules represented 70-90% of the total surface bound C3b molecules. B. Western blot analyses of biotinylated and selfamplified C3b on streptavidin beads. As described in Barrio et al, we found evidence for covalent attachment of C3b molecules on top of other C3b (covalent C3b multimers). C. C5 convertase activity on C3b beads and serum-amplified C3b beads (with equal amounts of C3b per bead). Data of three independent experiments, presented as means ± SD. Figure S4. Quantification of C3b-biotin bound to tosyl streptavidin beads ('biotinylated C3b) versus tosyl-activated beads ('random C3b'). C3b levels were detected by flow cytometry after incubation of beads with an antibody against C3b. (PDF 1672 kb) [file 12915_2015_203_MOESM1_ESM.pdf]

**Appendix: "Molecular insights into the surface-specific arrangement of complement C5 convertase enzymes" by Berends et al.**

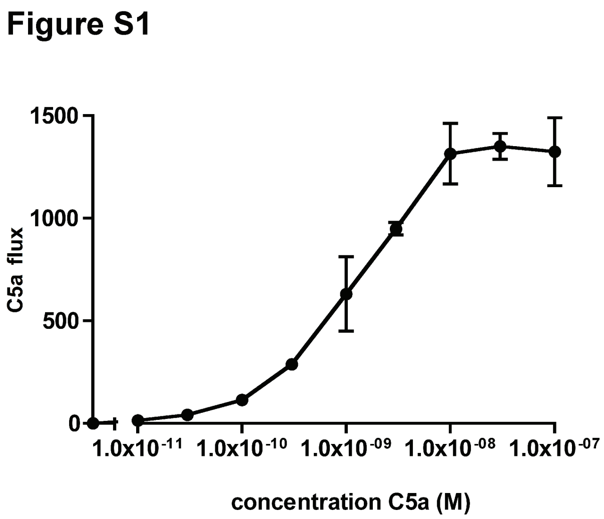


**Figure S1.** C5a standard curve for calcium mobilization assay. Different concentrations of purified C5a were added to U937-C5aR cells as stimulus to detect calcium mobilization. Data represent mean values of fluorescence after stimulus, subtracted by the mean fluorescence before stimulus of two independent experiments (presented as means ± standard deviation (SD)).

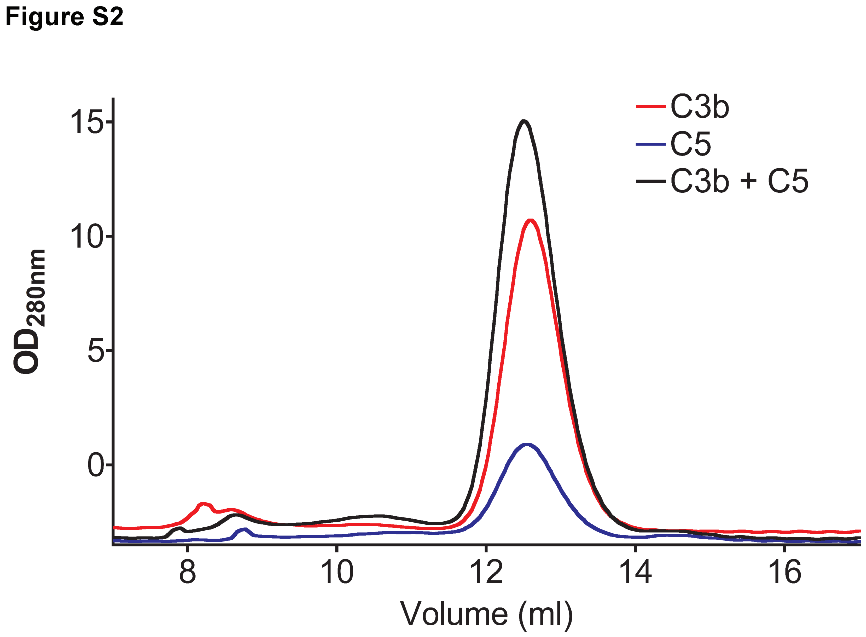


**Figure S2.** Size exclusion chromatography of soluble C3b and C5. C3b-biotin (300µg/ml), C5 (150µg/ml) or a mixture of both proteins at the same concentrations were incubated for 1 hour at 4°C in VBS with 2.5mM $MgCl_2$ and subsequently analysed on a Superdex 200 10/300 GL column (100µl injection volume). Representative chromatogram of three independent experiments.

**Figure S3**

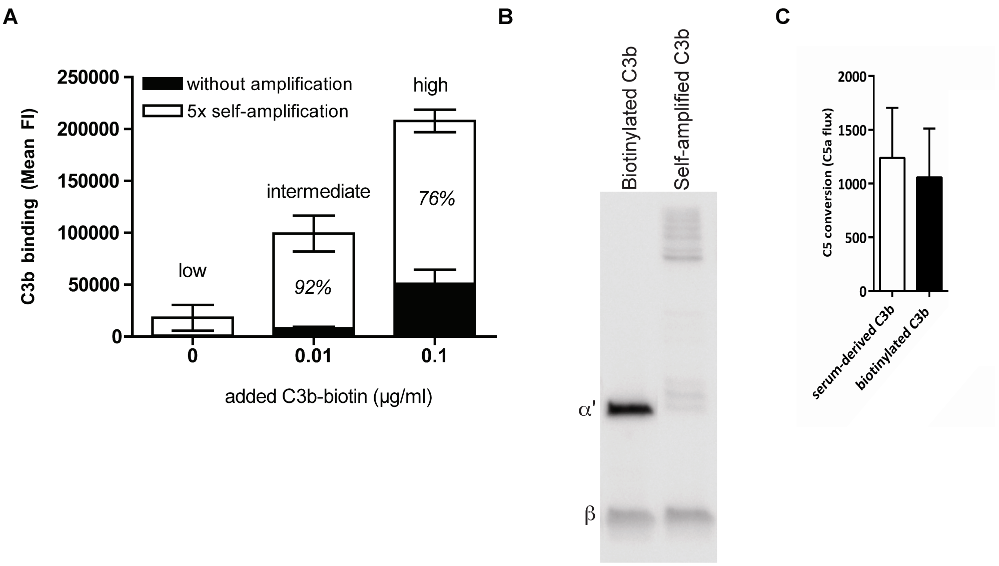


**Figure S3.** *A*. C3b quantification of self-amplified beads. C3b deposition was quantified using a FITC-conjugated C3 antibody and flow cytometry. Black bars indicate the initial biotinylated C3b on beads. White bars indicated the C3 fluorescence that was loaded via self-amplification after 5 rounds of incubation with FB, FD and C3. Total surface C3b levels were categorized in 'low', 'intermediate', and 'high' levels. 'Self' deposited C3b molecules represented 70-90% of the total surface bound C3b molecules. *B*. Western blot analyses of biotinylated and self-amplified C3b on streptavidin beads. As described in *Barrio et al*, we found evidence for covalent attachment of C3b molecules on top of other C3b (covalent C3b multimers). *C*. C5 convertase activity on C3b beads and serum-amplified C3b beads (with equal amounts of C3b per bead). Data of three independent experiments, presented as means ± SD.

**Figure S4**

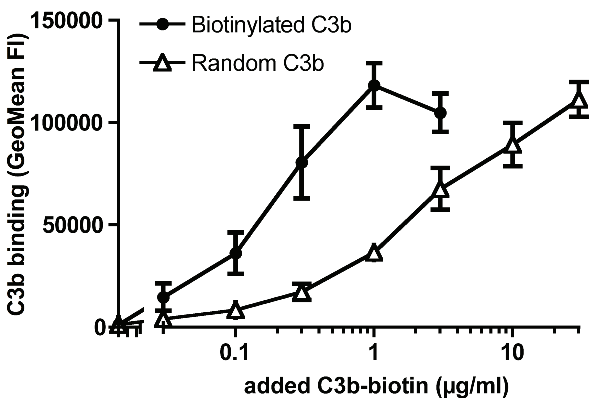


**Figure S4.** Quantification of C3b-biotin bound to tosyl streptavidin beads ('biotinylated C3b) versus tosyl-activated beads ('random C3b'). C3b levels were detected by flow cytometry after incubation of beads with an antibody against C3b.
